# Effects of subanesthesia dose S‐ketamine induction on postoperative psychiatric complications after gynecological surgery

**DOI:** 10.1002/ibra.12039

**Published:** 2022-05-10

**Authors:** Fan Zhang, Jun Ding, Man Luo, Hao‐Hua Luo, Xiao‐Lin Sun, Xu Fang, Lei Chen, Jun Tao, Zhao‐Qiong Zhu

**Affiliations:** ^1^ Department of Anesthesiology Affiliated Hospital of Zunyi Medical University Zunyi Guizhou China; ^2^ Department of Anesthesiology Qian Xi Nan People's Hospital Qianxinan Guizhou China; ^3^ Department of Anesthesiology Tongren Municipal People's Hospital Tongren Guizhou China

**Keywords:** gynecological surgery, induction, pain relief, psychiatric complication, S‐ketamine

## Abstract

Ketamine may become an important drug for multimodal analgesia regime again because of its strong analgesic effects and retaining the advantage of spontaneous breathing. The present study was designed to explore the influences of different dosages of S‐ketamine anesthesia induction regimes on psychiatric complications and postoperative prognosis in patients undergoing gynecological operations. In this prospective, triple‐blinded, randomized, controlled study, patients undergoing elective gynecological surgery were randomized to one of three treatment groups: low‐dose S‐ketamine (LDSK) group (a 0.3 mg/kg bolus for anesthesia induction), minimal‐dose S‐ketamine (MDSK) group (a 0.2 mg/kg bolus for anesthesia induction), and placebo (CON) group (a saline bolus for anesthesia induction). The main outcome measures were as follows: intraoperative vital signs, extubation time, anesthesia recovery time and postanesthesia care unit (PACU) stay duration, incidence of psychiatric complications, Ramsay sedation scale (RSS) 1, 2, 24, and 48 h, postoperatively, and overall prognosis. One hundred and eighty female participants were finally included in this study from April 2021 to December 2021. Significant differences were not observed in age, height, weight, American Society of Anesthesiologists physical status classification, or history of mental illness between the groups. No statistically significant differences were discovered with regard to intraoperative vital signs, extubation time and PACU stay duration, incidence of psychiatric complications, and RSS scores at 1, 2, 24, and 48 h postoperatively in the three groups. However, the visual analog scale (VAS) scores of the CON group at 10 min after extubation and at the time point leaving PACU were much higher than that of the LDSK and MDSK groups. The VAS scores at 48 h after surgery in the MDSK group were also lower than that of the CON group and the CON group had received more analgesic drug treatment in the surgical wards consequently. Postoperative nausea and vomiting (PONV) occurrence at 24 and 48 h, postoperatively, increased sharply in the CON group than in the other two experimental groups, which led to an increase in the use of postoperative antiemetic drugs in this group. According to the postoperative satisfaction survey, patients in the CON group had lower medical satisfaction. Our data demonstrate that a small dosage of S‐ketamine anesthesia induction can reduce postoperative pain and the incidence of PONV without increasing hemodynamic fluctuations or psychiatric complications.

## Graphical abstract

This article evaluated the effect of the new‐type drug S‐ketamine on the cognitive function of gynecological patients through a randomized controlled clinical study, hoping to optimize clinical anesthesia management.

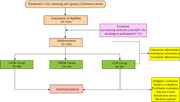

## 1 INTRODUCTION

Opioids are the most widely administered analgesics in clinical practice. The anesthetic and analgesic potency of opioids is beyond doubt. However, many studies have demonstrated that opioids are not conducive to the prognosis of surgical patients.^1^, ^2^ Evidence‐based medicine suggests that multimodal analgesia schemes and opioid‐less anesthesia should be enhanced in clinical work.^3^ Ketamine, which was first introduced to the anesthesia field in the 1970s, has regained broad interest in perioperative medicine within the last few years.^4^ A broad evidence base now confirms that the perioperative administration of ketamine may decrease opioid consumption. Unfortunately, the use of these drugs is limited because of their potential hemodynamic effects and psychiatric complications. S‐ketamine is a new‐type drug to Chinese anesthesiologists, which is a dextral body of ketamine. In the previous research on adults, it was declared that S‐ketamine is superior to the traditional racemic ketamine in terms of greater potency. To reduce the risk for potential neuropsychiatric complications, however, the administered dose of S‐ketamine has been limited to a maximum intraoperative bolus of 1 mg/kg, which is considered a low‐dose regimen.^5^ A intraoperative bolus of 0.2 mg/kg for anesthesia induction, which is considered a minimal‐dose regimen.^6^ No clear optimal dose of S‐ketamine during anesthesia induction has been detected to date. We hypothesized that if S‐ketamine was partially applied in the multimodal anesthesia, its dosage, which was even lower than the dosage with unanimous consent in the medical field, could achieve better results in reducing postoperative complications. Thus we compared the effects of S‐ketamine induction regimens at different dosages on opioid consumption, psychiatric complications, and prognosis of patients to provide further experiences for clinical management.

## 2 MATERIALS AND METHODS

### 2.1 Patients selection and inclusion process

After receiving approval to carry out the study from the institutional ethics review board of Zunyi Medical University (KLL‐2020‐049), eligible subjects were contacted 1 week before the operation and signed informed consent after they had been fully informed about the study process. The trial process had been registered at chictr.org.cn (no.ChiCTR2100046703) before being launched. Patients were scheduled for surgical treatment from April 2021 to December 2021. Inclusion criteria: patients undergoing elective gynecological surgery, aged between 18 and 65 years old, body mass index between 18 and 30 kg/m^2^, American Society of Anesthesiologists (ASA) physical status class Ⅰ–Ⅱ, operative duration between 0.5 and 3 h. Exclusion criteria: severe liver or kidney dysfunction, severe cardiovascular diseases, intracranial hypertension and central nervous system diseases (schizophrenia, mania, bipolar disorder, insanity, etc.), hyperthyroidism, pregnancy and lactation, history of drug or alcohol abuse, present or past psychotic disorders. Elimination criteria: subjects withdraw informed consent at any time; subjects were found unqualified after random division; ingestion of medication (other than the study protocol) within 48 h of surgery, or subjects might benefit less than the risk due to any clinical adverse event.

### 2.2 Study grouping scheme

All qualified patients were randomly divided into one of the three treatment groups, as follows:

Low‐dose S‐ketamine (LDSK) group: a 0.3 mg/kg intravenous (i.v.) bolus of S‐ketamine was given for anesthesia induction.

Minimal‐dose S‐ketamine (MDSK) group: a 0.2 mg/kg intravenous (i.v.) bolus of S‐ketamine was given for anesthesia induction.

Placebo (CON) group: the control group received 0.9% saline in the same volume as the above two groups for anesthesia induction.

### 2.3 Randomization and blinding

The study medication was prepared preoperatively using a computer‐generated randomization list, by an anesthetist who was excluded from any further involvement in the treatment or evaluation of patients. The syringes for the bolus injection were labeled with “Study Medication” and the randomization number of the patient in this study. All involved stuffs were strictly kept unknown about group allocation beforehand.

### 2.4 Anesthesia induction management

There was no preoperative medication in this study. After anesthesia preparation, the researchers obtained the results of a randomized grouping of patients according to the random number method. Anesthesia was induced and maintained following our institutional standards: propofol (BB210807; Enhua) 2.5 mg/kg, sufentanil (11A02091; Yichang Humanwell) 0.3 μg/kg, rocuronium (EA2147; Xianju) 0.6 mg/kg. After injection of the above drugs, patients in the three groups received different doses of S‐ketamine (210219BL; Hengrui) or a normal saline placebo injection according to the grouping scheme. Endotracheal intubation was performed after the depth of anesthesia was reached. After endotracheal intubation, mechanical ventilation was performed immediately: tidal volume (TV) 8–10 ml/kg, respiratory rate (RR) 12–20 times/min, and the PetCO_2_ was maintained between 35 and 45 mmHg. Intravenous infusion of propofol (BB210807; Enhua) 4–8 mg/kg/h and remifentanil (6180411; Yichang Humanwell) 6–10 μg/kg/h was used for anesthesia depth maintenance. Rocuronium (EA2147; Xianju) was added intermittently according to the operation requirements to maintain muscle relaxation. It was forbidden to use inhaled anesthetics or local anesthetics during the anesthesia process in this study. Invasive hemodynamic monitoring was selected according to the surgical routine. The infusion volume was adjusted according to the monitoring index during operation. After the operation, all subjects were transferred to the Postanesthesia Care Unit (PACU) for resuscitation.

### 2.5 Patient‐controlled intravenous analgesia and remedy management

Immediately at the end of the operation, the PCIA pump (WZ‐6523C4; Funiya) was attached to the patients' intravenous cannula in a unified way for all three groups: sufentanil (11A02091; Yichang Humanwell) (2 μg/kg) and metoclopramide (62009041; Suicheng) (40 mg) were jointly configured into 100 ml elastomeric pumps. PCIA lasted until 48 h after the operation. When required, postoperative pain was treated with 40 mg paresibuna (F2102032; Aosaikang). If the patient complained of pain again, sufentanil (11A02091; Yichang Humanwell) 5 μg was used as a pain remedy. To ensure the quality of research, all analgesia schemes for perioperative patients were determined by anesthesiologists.

### 2.6 Study outcomes

The primary outcome endpoints were the intraoperative hemodynamic signs, extubation time, PACU stay time, incidence of psychiatric complications, and Ramsay sedation scale (RSS) scores at 1, 2, 24, and 48 h postoperatively. Our secondary outcomes included perioperative opioids consumption, visual analog scale (VAS) scores 10 min after extubation, at the time point leaving PACU, and at 1, 2, 24, and 48 h postoperatively, complications and prognosis, duration of hospital stay, satisfaction survey results, and also medical expenses.

### 2.7 Sample size calculation

When designing the study, no published literature was available to estimate the effects of different doses of S‐ketamine on psychiatric complications of patients. We used the PASS 20.0 software (NCSS, LLC) for calculation, setting a significance level of 0.05 and *β* to 20%, we estimated that at least a total sample size of 90 patients would be required (30 in each group). To further reduce the bias, a population of 180 patients was considered to be included in this study.

### 2.8 Statistical analysis

Data were tested for normality by using the Kolmogorov–Smirnov test. Normally distributed variables are expressed as the means ± standard deviations, and abnormally distributed variables are expressed using the median (minimum, maximal). Categorical variables are expressed as numbers (percentages). Differences between groups were calculated by analysis of variance or the Kruskal–Wallis test, as appropriate. Categorical variables were analyzed using the *χ* ^2^ test or Fisher's exact test. Data were analyzed using SPSS (version 24.0; SPSS Inc., IBM). All statistical tests were two‐tailed, and a *p* < 0.05 was defined as statistically significant.

## 3 RESULTS

**Figure 1:**
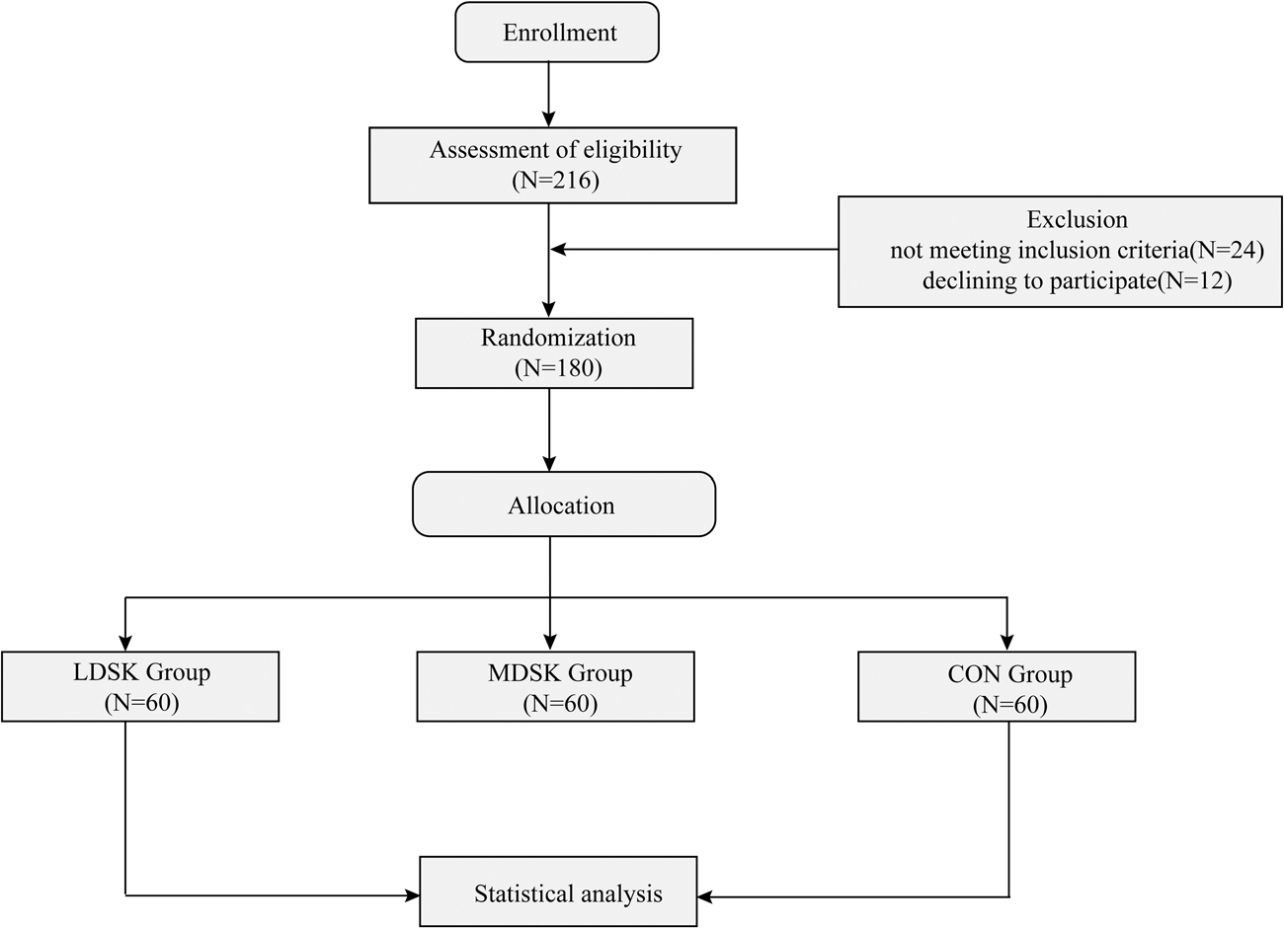
Summary chart of the research process. CON, Placebo; LDSK, low‐dose S‐ketamine; MDSK, minimal‐dose S‐ketamine

Two hundred and sixteen patients were assessed for eligibility from April 2021 to December 2021, and 36 patients were excluded from this study for failing to meet the inclusion criteria. Of the 180 patients included and randomly assigned in this study, no participants were excluded for violation of study protocols or any other reasons. Eventually, 60 patients entered the analysis stage in each group. The complete research process is summarized in Figure 1.

### 3.1 Baseline and surgery information

Significant differences were not detected in age, height, weight, ASA physical status classification, or history of mental illness between the groups. Likewise, no significant differences were found in the preoperative diagnosis, the laboratory test results, duration of anesthesia, operation mode, length of surgery (skin incision to closure), and intraoperative volume between the groups. The baseline characteristics of the three groups were tested to be comparable (Table 1, *p* > 0.05).

**Table 1:** Comparison of basic data between groups

Baseline indicators	LDSK group (*n* = 60)	MDSK group (*n* = 60)	CON group (*n* = 60)	*p* value
Age (years)	37.8 ± 11.1	39.4 ± 10.2	38.7 ± 9.8	0.659
Height (cm)	158.1 ± 6.4	157.3 ± 6.6	157.8 ± 5.6	0.769
Weight (kg)	56.6 ± 8.4	55.6 ± 5.9	57.5 ± 7.6	0.481
BMI(kg/m^2^)	22.6 ± 2.7	26.4 ± 2.4	23.0 ± 2.6	0.377
ASA classification (*n* [%])				0.788
Ⅰ	20 (33.3)	17 (28.3)	17 (28.3)	
Ⅱ	40 (66.7)	43 (71.7)	43 (71.7)	
Diagnosis (*n *[%])				0.957
Ovarian lesions	20 (33.3)	19 (31.7)	21 (35.0)	
Uterine lesions	35 (58.4)	37 (61.7)	36 (60.0)	
Vulvar lesions	5 (8.3)	4 (6.6)	3 (5.0)	
Duration of anesthesia (min)	101.2 ± 45.0	125.1 ± 68.5	124.5 ± 48.8	0.027
Duration of surgery (min)	75.5 ± 38.9	92.7 ± 47.0	97.1 ± 43.6	0.045
Intraoperative volume				
Liquid volume (ml)	1095.0 ± 359.7	1167.0 ± 525.6	1179.0 ± 398.2	0.520
Urine volume^a^ (ml)	186.3 (0–800)	219.3 (0–1300)	219.8 (0–500)	0.558

*Note*: Data are presented as the mean ± standard deviation or percentage.

Abbreviations: CON, Placebo; LDSK, low‐dose S‐ketamine; MDSK, minimal‐dose S‐ketamine.

^a^ Nonnormal distribution data are presented as median (minimum, maximal).

### 3.2 Evaluation of perioperative hemodynamics trends

**Figure 2:**
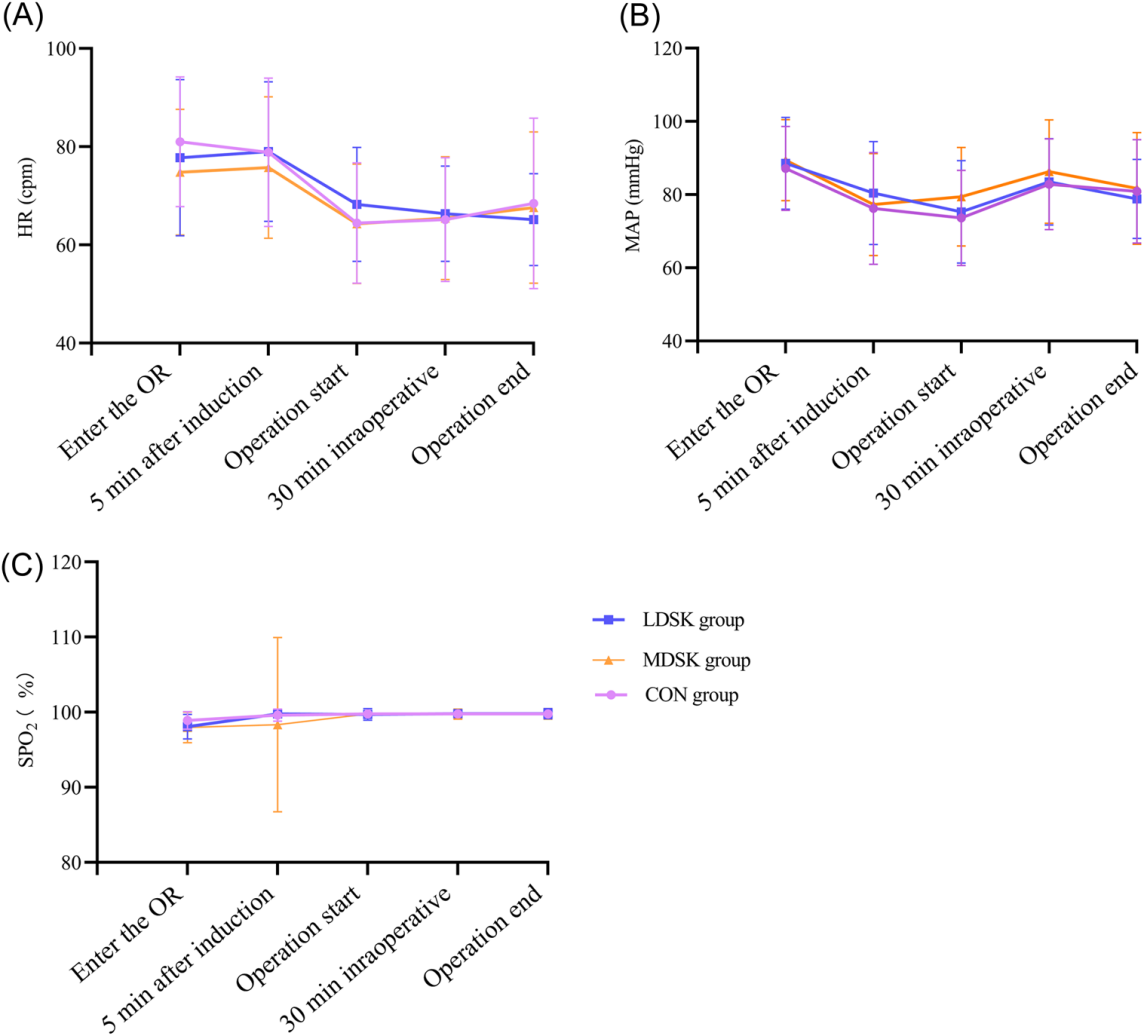
Comparison of hemodynamic changes between groups. (A, B, C) Comparison of heart rate changes, mean arterial pressure, pulse oxygen saturation. The data are expressed as mean ± standard deviation. *p* > 0.05. CON, Placebo; LDSK, low‐dose S‐ketamine; MDSK, minimal‐dose S‐ketamine [Color figure can be viewed at wileyonlinelibrary.com]

In this study, heart rate (HR), mean arterial pressure (MAP), and pulse oxygen saturation (SpO_2_) between groups at different observational time points were similar to each other. Severe hypertension or hemodynamic fluctuations were not found postoperatively (Figure 2, *p* > 0.05).

### 3.3 Evaluation of anesthesia resuscitation time, perioperative sedative effects, and psychiatric complications

**Figure 3:**
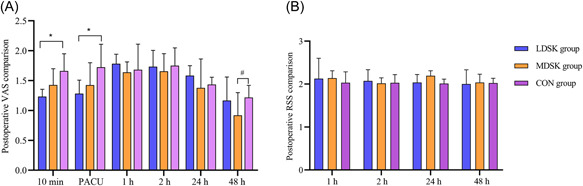
Comparison of VAS and RSS between groups. (A) Postoperative VAS of three groups. (D) Postoperative RSS of three groups. The data are expressed as mean ± standard deviation. *p < 0.05, ^#^ *p* < 0.05. CON, Placebo; LDSK, low‐dose S‐ketamine; MDSK, minimal‐dose S‐ketamine; RSS, Ramsay sedation scale; VAS, visual analog scale [Color figure can be viewed at wileyonlinelibrary.com]

No obvious delay was found in the total extubation time or PACU discharge time between the groups (Table 2, *p* > 0.05). Significant statistical differences were not observed with regard to RSS scores at 1, 2, 24, and 48 h, postoperatively (Figure 3B, *p* > 0.05). The details of psychiatric complications over time are presented in Table 3. Complications such as drowsiness, dysphoria, somnolence, dizziness, headache and delirium occurred in different groups. Dizziness was the most common psychiatric complication in the three groups. The LDSK, MDSK, and CON groups reported 6, 5, and 4 cases of dizziness respectively during the study process. However, no differences were reported with regard to perioperative psychiatric complications between the groups (Table 3, *p* > 0.05).

**Table 2:** Drug consumption and PACU resuscitation between groups

Outcome measures	LDSK group (*n* = 60)	MDSK group (*n* = 60)	CON group (*n* = 60)	*p* value
Intraoprative opioids dosage				
Sufentanil (μg)	16.9 ± 2.2	17.3 ± 2.4	17.2 ± 2.5	0.689
Remifentanil (mg)	0.8 ± 0.7	0.9 ± 0.7	1.0 ± 0.6	0.282
Intraoprative anesthetics dosage				
Propofol (mg)	519.7 ± 377.4	708.7 ± 352.1	671.3 ± 285.3	0.065
Rocuronium (mg)	44.2 ± 15.3* ^,^ **	52.0 ± 7.4	52.7 ± 7.8	0.001
Extubation time (min)	23.3 ± 12.9	23.6 ± 14.4	21.1 ± 14.4	0.568
PACU stay time (min)	51.1 ± 12.7	51.9 ± 17.3	49.2 ± 15.5	0.607
PACU drug consumption (*n *[%])				0.586
Paresibuna	0	0	4 (6.7)	
Neostigmine + Atropine	8 (13.3)	12 (20)	4 (6.7)	
Metoclopramide	0	0	1 (1.6)	
Ward drug consumption (*n *[%])				<0.001
Paresibuna	0	2	4 (6.7)	
Metoclopramide	0	2	11 (18.3)	
Analgesic pump pressing^a^ (times) (h)				
2	0 (0,2)	0 (0,2)	0 (0,3)	0.050
24	0 (0,6)**	0 (0,6)**	0 (0,12)	0.004
48	0 (0,6)	0 (0,2)	0 (0,3)	0.080

Abbreviations: CON, Placebo; LDSK, low‐dose S‐ketamine; MDSK, minimal‐dose S‐ketamine.

^a^ Non normal distribution data are presented as median (minimum, maximal).

* *p* < 0.05 compared with the MDSK group

** *p* < 0.05 compared with the CON group.

**Table 3:** Comparison of psychiatric and prognostic indicators between groups

Outcome measures	LDSK group (*n* = 60)	MDSK group (*n* = 60)	CON group (*n* = 60)	*p* value
Psychiatric complications (*n *[%])				0.586
Drowsiness	1 (1.6)	1 (1.6)	1 (1.6)	
Dysphoria	0	1 (1.6)	0	
Somnolence	0	1 (1.6)	0	
Dizziness	6 (10)	5 (8.3)	4 (6.7)	
Headache	1 (1.6)	4 (6.7)	2 (3.3)	
Delirium	1 (1.6)	1 (1.6)	1 (1.6)	
PONV occurrence (*n* [%]) (h)				
2	6 (10)	8 (13.3)	11 (18.3)	0.414
24	20 (33.3)	16 (26.7)	30 (46.7)* ^,^ **	0.024
48	21 (35)	17 (28.3)	31 (48.3)* ^,^ **	0.026
Total hospital stay (day)	8.6 ± 2.7	8.5 ± 2.1	8.5 ± 2.2	0.816
Postoperative hospital stay (day)	4.9 ± 1.5	4.9 ± 1.1	4.9 ± 1.7	0.995
Satisfaction survey (*n* [%])	58 (96.7)	58 (96.7)	50 (83.3)* ^,^ **	0.001
Medical expense (10,000 yuan)	1.3 ± 0.2	1.4 ± 0.2	1.3 ± 0.5	0.805

Abbreviations: CON, Placebo; LDSK, low‐dose S‐ketamine; MDSK, minimal‐dose S‐ketamine.

* *p* < 0.05 compared with the LDSK group

** *p* < 0.05 compared with the MDSK group.

### 3.4 Comparison of pain, opioids consumption, and PONV control

Compared with the CON group, the VAS scores at 10 min after extubation and at the time point leaving PACU in the LDSK and MDSK groups were significantly lower. A significant difference was also reported between the MDSK and CON group at 48 h during rest, postoperatively (Figure 3A, *p* < 0.05). The anesthesia drug consumption had no significant difference between groups at PACU (Table 2, *p* > 0.05), but with a difference in the surgical wards (Table 2, *p* < 0.05). The CON group was reported to have the highest analgesic pump pressing times within 24 h after the operation. The incidence of PONV after 24 and 48 h after operation in the CON group was much higher, as nearly half (48.3%) of the patients in this group developed PONV within 48 h after the operation; the patients also experienced more severe postoperative PONV and drug antiemetic therapy (Table 3, *p* < 0.05).

### 3.5 Assessment of secondary endpoints

There was no significant difference in postoperative fluid drainage amount, drainage tube extraction time, wound healing, bed rest time, total hospital stay, postoperative length of stay, and medical expenses between the two groups (Table 3, *p* > 0.05). With regard to the medical satisfaction survey, compared with the CON group, patients in the S‐ketamine group experienced higher medical satisfaction (96.7%) (Table 3, *p* < 0.05).

## 4 DISCUSSION

Ketamine, the only intravenous general anesthetic with analgesic effects, was widely used in clinical anesthesia many years ago, especially in short‐term surgical anesthesia for children. In the past, ketamine was reported to have a great impact on hemodynamics with increased secretion and potential postoperative psychiatric complications, which severely limited its clinical application.^7^, ^8^ S‐ketamine, a synthetic drug of ketamine dextroisome, is a new drug for Chinese anesthetists but there were few medical reports about it. It mainly acts on *N*‐methyl‐d‐aspartic acid (NMDA) receptors, inhibiting the activation of glutamine on this receptor. The block of NMDA is time‐dependent and stimulation‐frequency‐dependent, so as to weaken the activity of neurons and achieve anesthetic and analgesic effects. According to previous pharmacological research, The affinity of NMDA receptors and u receptors of the S‐enantiomer was reported to be four times more potent than that of the tranditional levoisome, the affinity of M acetylcholine receptors of S‐enantiomer has been suggested to be two times more potent than the previous tranditional levoisome.^9^, ^10^ The routine anesthesia induction dose of S‐ketamine is 0.5–1 mg/kg. It is reported that patients still have a certain proportion of postoperative complications such as delirium at this dose. Therefore, this study discusses the effects of subanesthesia dose S‐ketamine on patients through the set of low‐dose and minimal‐dose groups. Low‐dose racemic ketamine is defined as an intravenous bolus dose of less than 1 mg/kg. Theoretically, at a low dose, S‐ketamine will preferentially bind to postsynaptic NMDA receptors in the spinal dorsal horn rather than to receptors in the brain. Therefore, a low dose of S‐ketamine has a lower risk of mental side effects. In this study, we designed a conservative treatment regime to reduce the risk of mental side effects triggered by the minimum dose of S‐ketamine.^11^ Little information is available about the application of S‐ketamine in women undergoing gynecological surgery, in particular when they are administered as a subanesthesia treatment. The efficacy of these drugs in this context needs to be further investigated.

Ketamine drugs indirectly excite the cardiovascular system by exciting the sympathetic nerve center, which is mainly manifested in the increase of HR, blood pressure, cardiac index, and peripheral vascular resistance. At the same time, it can also cause the sympathetic ganglion to release norepinephrine.^12^ The results of the present study showed that the HR of all patients was stable. HR remained constant in all three groups, especially at 5 min after anesthesia induction. A similar pattern was observed in the change of systolic and diastolic arterial pressures and SpO_2_, perioperatively. No severe hypertension or tachycardia occurred in the two S‐ketamine groups during the study. The above results suggest that a small dose of S‐ketamine induction is safe and reliable in maintaining hemodynamic stability in elderly women. Moreover, during the induction of general anesthesia, general anesthetics including propofol have strong myocardial inhibition and vasodilation effects, which is unfavorable to advanced aged gynecological patients. The sympathetic excitation property of S‐ketamine avoided the occurrence of cardiovascular and cerebrovascular accidents related to severe hypertension or hypotension after induction. Small dose S‐ketamine used for general anesthesia induction is recommended by this study.

Ketamine has an excitatory effect on the limbic system of the brain, resulting in the separation of consciousness and feeling, so there are many adverse reactions. The main manifestations are mental and nervous system reactions, such as illusion, schizophrenia, irritability, disorientation, cognitive impairment, delirium, moderate muscle tension, and tremor.^13^ These adverse reactions can occur after administration or during postoperative recovery. The greater the dose, the more significant the effects. These adverse reactions greatly limit its further clinical use. In this study, the treatment of LDSK did not cause a delay in anesthesia awakening, and the awakening quality of the three groups was good. The extubation time of the three groups was all less than 30 min and the PACU recovery time was less than 1 h. During the perioperative period, the RSS scores of the three groups were mainly within 2 to 3, indicating the level of mild sedation. The depth of postoperative sedation of the three groups was moderate, which, in turn, ensured medical safety in the surgical wards.

The postoperative psychiatric complication is a major concern in perioperative medicine, and ketamine has been associated with a prolonged time to resumption of mental orientation. Psychodysleptic symptoms are well‐described side effects of ketamine. In the previous literature, the most reported postoperative complication related to ketamine was postoperative delirium. However, in our population, the risk for psychiatric complications was comparable, whereas there were no significant differences between all groups, which is in accordance with the current literature indicating that psychomimetic effects of S‐ketamine are dose‐dependent.^4^, ^14^, ^15^ The most common postoperative psychiatric complication of the three groups was dizziness. It cannot be denied that this complication was partly related to the special surgery position and cerebral blood flow changes during gynecological endoscopic surgeries. Therefore, further research should be carried out to exclude the effects of different surgical positions on patients' cognitive function.

Our data showed that minimal‐dose 0.2 mg/kg S‐ketamine induction was comparably effective to conventional LDSK in reducing postoperative pain. Moreover, pain scores differed significantly between the three groups at postoperative short‐term time points, and this analgesic effect even lasted until 48 h after the operation. It is known that tissue damage may trigger altered processing of noxious stimuli so that perception of pain persists even after cessation of nociceptive input. This is referred to as central sensitization and represents a mechanism of amplification of pain perception.^16^, ^17^, ^18^, ^19^ Due to the central role played by NMDA receptors in central sensitization, ketamine and S‐ketamine may be useful for their analgesic and anti‐hyperalgesic properties. In many clinical trials, ketamine isomers showed to reduce analgesic consumption when used as an adjuvant for pain treatment through various routes of administration.^20^, ^21^ The results suggest that even half of the routine dose of S‐ketamine induction can also significantly improve postoperative pain.

Female patients are associated with a high risk of PONV. The use of opioids may activate NMDA receptors, leading to acute opioid tolerance and postoperative hyperalgesia, resulting in increased postoperative pain.^22^ In this study, there was no significant difference in the dosage of narcotic drugs, especially opioid consumption among the three groups in the operating room (OR). However, with the decreased dose of S‐ketamine, the dosage of opioids increased in different experimental groups in the surgical wards, resulting in an obviously increased incidence of postoperative PONV within 48 h after operation. In addition, more patients of the CON group pressed the analgesic pumps for pain relief 24 h after the operation according to follow‐up. This also highlights the characteristics of NMDA blocker S‐ketamine in inhibiting hyperalgesia and the consumption of opioids.

The dosage of rocuronium consumption in the CON group was also much higher than that in the other two groups. Ledowski et al.^2^ compared the effects of S‐ketamine on intubating conditions with fentanyl. The combination of propofol and S‐ketamine for anesthesia induction achieved the best intubating conditions with the least rocuronium consumption. The above research results are highly consistent with our results. Tracing the reasons, S‐ketamine can ensure normal cardiac output (CO) and blood supply during general anesthesia and sharply reduce the dose of combined drugs. According to the postoperative follow‐up, the patients in the CON group experienced more postoperative pain and pressing times of analgesic pumps than the other two groups, which was also an important reason for a high incidence of PONV in this group. From the results of this study, we believe that S‐ketamine has application advantages in multimodal analgesia, especially for postoperative PONV control. Previous studies regarding cesarean section have suggested that LDSK can not only relieve pain but also play an antidepressant role, which may also be the reason for the low incidence of PONV and high satisfaction rate of two S‐ketamine groups. Its antidepressant effect is highly related to the activation of the α‐amino‐3‐hydroxy‐5‐methyl‐4‐isoxazole‐propionicacid (AMPA) receptor.^23^, ^24^ According to the questionnaire, the main causes of dissatisfaction were pain and a high incidence of PONV, which also suggests that our anesthesia decision‐making still needs to be improved.

In this study, no obvious difference was detected between the two S‐ketamine subgroups in pain control and prevention of mental complications. The prognosis results are similar, suggesting that a 0.2 mg/kg injection is enough for balanced anesthesia induction.

The major limitation of our study is that we did not include all possible dose study groups, which would represent an interesting topic for further research. In our population, however, no significant differences were found in intraoperative opioid consumption between groups. Therefore, we conclude that a single injection of S‐ketamine has limited effects on saving opioid consumption.

In conclusion, a minimal‐dose S‐ketamine regimen is effective in reducing postoperative pain following gynecological surgery and can reduce the risk of postoperative psychiatric complications when compared with a low‐dose regimen. We therefore suggest that subanesthesia S‐ketamine 0.2 mg/kg be used as a low‐risk component of balanced perioperative analgesia.

## AUTHOR CONTRIBUTIONS

Fan Zhang contributed to study design, research implementation, and writing. Jun Ding contributed to the patients' inclusion process. Man Luo, Hao‐Hua Luo, and Xiao‐Lin Sun contributed to participants' follow‐up in PACU and wards. Xu Fang, Lei Chen, and Jun Tao contributed to the statistical analysis. Zhao‐Qiong Zhu contributed to picture making, adverse event reports, and study quality control. All authors revised and approved the final article version, and agreed to be accountable for all aspects of the work.

## CONFLICTS OF INTEREST

Zhao‐Qiong Zhu is a reviewer for Ibrain but is not involved in the peer review process of this manuscript. Other authors declare no conflicts of interest.

## TRANSPARENCY STATEMENT

The author confirms that this manuscript is an honest, exact, and transparent description of the reported research. There is no omission or concealment of any important aspect in the study or any differences from the planned study before the start.

## ETHICS STATEMENT

Ethics Committee of Affiliated Hospital of Zunyi Medical University approved all experiments on the involved patients (no. KLL‐2020‐049). This trial had been registered at chictr.org.cn (no. ChiCTR2100046703) and was conducted strictly in accordance with the Declaration of Helsinki. Written consent to participate was signed for all the patients. Eligible patients were contacted and informed about the study.

## Data Availability

The data that support the findings of this study are available on request from the corresponding author. The data are not publicly available due to privacy or ethical restrictions.
